# How Live Performance Moves the Human Heart

**DOI:** 10.1371/journal.pone.0154322

**Published:** 2016-04-22

**Authors:** Haruka Shoda, Mayumi Adachi, Tomohiro Umeda

**Affiliations:** 1 Department of Psychology, Hokkaido University, Sapporo, Hokkaido, Japan; 2 Nara Medical University, Kashihara, Nara, Japan; University of Zurich, SWITZERLAND

## Abstract

We investigated how the audience member’s physiological reactions differ as a function of listening context (i.e., live versus recorded music contexts). Thirty-seven audience members were assigned to one of seven pianists’ performances and listened to his/her live performances of six pieces (fast and slow pieces by Bach, Schumann, and Debussy). Approximately 10 weeks after the live performance, each of the audience members returned to the same room and listened to the recorded performances of the same pianists’ via speakers. We recorded the audience members’ electrocardiograms in listening to the performances in both conditions, and analyzed their heart rates and the spectral features of the heart-rate variability (i.e., HF/TF, LF/HF). Results showed that the audience’s heart rate was higher for the faster than the slower piece only in the live condition. As compared with the recorded condition, the audience’s sympathovagal balance (LF/HF) was less while their vagal nervous system (HF/TF) was activated more in the live condition, which appears to suggest that sharing the ongoing musical moments with the pianist reduces the audience’s physiological stress. The results are discussed in terms of the audience’s superior attention and temporal entrainment to live performance.

## Introduction

Live music performance offers a special experience that is impossible through speakers or a headphone. This unique experience, often described as “communication” or “interaction”, has been studied empirically. For example, “visual” aspects of live performance, even presented as a video without sound, help the audience differentiate the performer’s intended levels of expressivity [1] and emotions [2, 3], enhancing the observer’s physiological reactions [4]. We investigated the effect of live performance on the audience’s physiology, not through a video but through a live context. By doing so, we tapped into a biological aspect of a performer-to-audience communication.

Since the pioneering study by Krumhansl [5], researchers have investigated the psychophysiological responses in listening to a recorded music, particularly with regard to the listener’s emotional experiences [6]. Previous studies used a number of parameters such as heart rate (electrocardiogram), sweat (electrodermal activity), skin temperature, muscle tension (electromyogram), and salivary cortisol, which increase in accordance with the listener’s experience of emotional arousal [6, 7]. Such responses in the autonomic nervous system can be explained by the brain activation. Blood and Zatorre [8] explored the listener’s heart rate, muscle tension, and respiration rate, as well as the cerebral brain activity by using positron emission tomography (PET), in experiencing “chills” (i.e., “shivers-down-the-spine”). They showed that the cerebral blood flow involved in reward/motivation are activated in experiencing chills (e.g., ventral striatum, midbrain, amygdala, orbitofrontal cortex, ventral medial prefrontal cortex), resulting in the activation of the autonomic (particularly sympathetic) nervous system.

The listener’s physiological reactions in watching a video recording of the performance have also been explored. Chapados and Levitin [4] measured the electrodermal activities (EDA) while listeners (or observers) were exposed to a video of a clarinet player in three modalities: audition-only, vision-only, and their combination. Higher EDAs were evident in the bimodal than the unimodal condition, suggesting that listening to music while watching a performer may facilitate the “rewarding” functions in humans. According to social psychologists, however, the presence of others generally decreases physiological arousal. For example, the spontaneous presence of others attenuates the physiological reactivity (i.e., heart rate, blood pressure) in performing a cognitive task, suggesting that social context can reduce stress during a particular task [9].

Would such stress-reduction effects be observable in an audience during a live music performance, in which the audience and the performer physically share time and space? Or would the audience’s physiology be aroused during live performance as found during the exposure to a performer through a recorded video? The purpose of our study was to test these hypotheses by comparing the audience’s physiological experiences in these two listening contexts: live (i.e., with performer) and recorded music contexts (i.e., through speakers without performer). More specifically, we assessed the audience’s heart rate and its variability across two ecologically valid listening contexts. It is common among music lovers that they went to a concert and later buy a live recording of the same concert that they enjoyed so much. We tried simulating this realistic situation in the study. The state-of-the-art technology enabled us to measure electrocardiograms of multiple audience members’ simultaneously without any cable connection to computers. Heart-rate measures, used most frequently of all physiological measures in music perception studies [6], enable us to assess both the sympathetic and the vagal nerve activities by computing spectral features of heart-rate time-series.

## Methods

### Participants

Seven pianists (2 men, 5 women, 24-40 years old, *M* = 30.57, *SD* = 6.46), who held a music degree in an undergraduate and/or graduate level, participated in this study. Each of 118 undergraduate and graduate students (53 men, 65 women) participated as an audience member for one of the live performances. Due to the limited number of heart-rate sensors (8-10 sensors for each session), the electrocardiograms were obtained from a total of 58 audience members, selected randomly from the participants who agreed with the physiological measurement. Of those, 21 data were excluded because the audience member failed to participate in both conditions (i.e., live and recorded contexts; see Procedure) or the insecure attachment of the sensor resulted in unreliable data. In the present study, we analyzed a sample of 37 audience members (16 men, 21 women, 18-26 years old, *M* = 20.59, *SD* = 2.06) who provided reliable data in all the conditions (i.e., six pieces in two listening contexts). The years of musical training (*N* = 37) outside of mandatory music education (i.e., 9 years of weekly classroom instruction including music appreciation) ranged from 0 to 19 years (*M* = 9.21, *SD* = 6.45); 25 had experienced piano performance for 1–19 years (*M* = 9.48, *SD* = 5.24). Written informed consent was obtained from every participant.

### Musical Pieces

We chose six pieces: b minor Prelude (Well-Tempered Clavier, Book I, No. 24, BWV869) and G major Prelude (Well-Tempered Clavier, Book II, No. 15, BWV884) by J. S. Bach, *Träumerei* (*Kinderszenen*, Op. 15-7) and *Aufschwung* (*Phantasiestüke*, Op. 12-2) by R. Schumann, *La fille aux cheveux de lin* (*Préludes* Book 2, L. 123-4) and Arabesque No. 1 (Two Arabesques for Piano, L. 66-1) by C. Debussy. We shall call these pieces “B24”, “B15”, “Dreaming”, “Soaring”, “Girl”, and “Arabesque”, respectively. We selected these three composers (i.e., Bach, Schumann, Debussy) based on our previous study [10], in which the majority of pianists chose these composers’ pieces as representatives of each historical period (i.e., Baroque, Romanticism, French Modernism). Based on the tempo instruction on the score, faster (B15, Soaring, and Arabesque) and slower (B24, Dreaming, and Girl) pieces were selected for each composer. B24 and Soaring are written in minor, and the rest in major.

### Apparatus

Experiments took place in a small auditorium with the maximum capacity of 114, equipped with a grand piano (GP-193, Boston). The piano was tuned professionally right before the live performance. The performances were recorded onto a multi-track recorder (R24, Zoom) using a microphone (NT4, Rode). A stereo speaker (WS-AT30, Panasonic), an amplifier (RX-V603, Victor), and a computer (MC505J/A, Apple) were used in playing the recorded sound to the audience in the recorded condition and the pink noise in resting phases of both conditions (see Procedure).

The audience’s electrocardiogram was measured by a heart-rate sensor (HRS-I, Win Human Recorder), attached on his/her left chest. The sensor is small and light enough (40 × 39 × 8 mm^3^, 14 g including a battery) not to disturb the audience’s listening experience. The sensors were synchronized with the timing of live performances by recording the onset time of electrocardiogram and the beginning of the first piece. The sampling frequency of electrocardiogram was 128 Hz and the data were recorded onto the sensor’s internal memory. We should acknowledge that this sampling frequency (i.e., 128 Hz) is less than the standard (> 250 Hz) [11]. However, the recent literature reports that the sampling frequency of 125 Hz is as valid as that of 1000 Hz [12], at least for healthy participants [13]. We also recorded the audience member’s bodily movement to examine whether the motion affect the electrocardiogram, by using the three-axis accelerometer equipped within the mobile sensor.

### Procedure

The experimental procedure in the present study was approved by the Committee for Research Ethics at the Graduate School of Letters, Hokkaido University, and the experiments conformed to the principles outlined in the Declaration of Helsinki. We adopted within-subject design as recommended by Potter and Bolls [14]: 2 (context)×3 (composer)×2 (tempo). All participants listened to six pieces (faster and slower pieces by Bach, Schumann, and Debussy) both on live (“live”) and from the speaker (“recorded”). Because we wanted to simulate a realistic situation in which one goes to a concert first and then encounters a recording of the exactly same live concert later, the order of the context was fixed: The audience participated in the recorded condition approximately 10 weeks after the live condition. We considered the insertion of 10 weeks to be enough to eliminate a possible mere-exposure effect (i.e., an effect that multiple exposures increase one’s preference), which can disappear in one month [15, 16].

In the live condition, we assigned 12 to 20 audience members to each of the live performance (of which 3-7 members were the target for the present study), so that they had a good view of the pianist. First, in order for the audience to be relaxed, they listened to a six-minute ocean-wave-like pink noise from the speakers that repeated five-second *crescendo* and five-second *decrescendo* (“resting phase”). The maximum sound level was 55.00 dB(A), which was measured at the center position of the audience by a sound-level meter (DT-8852, Mk Scientific). After the resting phase, the audience listened to the pianist’s live performances for six pieces. Two-minute break was inserted between the pieces. The order of the six pieces was determined by a block design. Namely, two pieces of one composer’s were performed first, followed by two pieces of another composer’s, and so on. In addition, the order of each composer’s two pieces was consistent based on the tempo. If the first piece was the faster one, the third and the fifth pieces were also the faster ones, and vice versa. Both the order of the composer and that of the tempo were counterbalanced among the pianists.

We instructed the audience to attend to both the sound and the pianist during the live performance. At the end of the live condition, the pianist and the audience provided demographic information by responding to a questionnaire, including the years of musical training and daily experiences in listening to and/or performing music.

Approximately 10 weeks after the live condition, participants came back to the same auditorium for the recorded condition. Following the resting phase, they listened to the same six performances, audio-recorded during the live condition, from the speakers. The sound level was checked by the aforementioned sound-level meter and adjusted if necessary so as to be consistent with the corresponding live performance. The presentation order was the same as the live condition, and the two-minute blank was inserted between the pieces.

The live and the recorded conditions lasted approximately 70 and 50 minutes, respectively. The live condition took longer because of the detailed explanation of the electrocardiogram sensor and a questionnaire for the participant’s background information.

### Parameters

First, we extracted the peak-to-peak intervals (RR interval) by the accessory software of HRS-I (Win Human Recorder), by which we calculated the mean heart rate (“HR”, 60/(mean RR intervals)). Then, the RR intervals were transformed into a continuous time-series with a sampling frequency of 4 Hz using cubic spline interpolation. We estimated the vagal and the sympathetic nerve activities by computing the Welch’s power spectrum densities at high-frequency (HF, 0.15–0.40 Hz), low-frequency (LF, 0.04–0.15 Hz), and total bands (TF, 0.04–0.40 Hz), respectively. The segment length of 512 samples (i.e., 128 s) with 50% overlap was used for the estimation because an approximately 2-minute recording is needed to address the LF component [11]. The very low frequency (VLF) (< 0.04 Hz) domain was not assessed in the present study because VLF computed from short-term recordings (≤ 5 minutes) are unreliable [11]. The HF/TF and the LF/HF ratios were obtained from these measures as indices of vagal nerve activity and sympathovagal balance, respectively [11, 17]. The LF/HF ratio was transformed into the natural logarithm in order for the data to be normally distributed. The vagal nerve activity functions as the defense reaction against an organism’s stress, or an index for stress reduction, represented by decreased heart rate and blood pressure. The sympathetic nervous system, often called as “fight and flight” nervous system, causes anxiety, manifested as shortness of breath and increased heart rate. The balance between these opposing neural mechanisms (sympathovagal balance) is often used as an index of mental stress [17, 18]. The parameters were calculated by Matlab 2015b (Mathworks).

## Results

### Preliminary Analyses

There are a few variables that could influence the audience’s heart-rate activities during two listening contexts. We conducted preliminary analyses for such variables.

The heart-rate sensor used in the present study was equipped with a function measuring the three-dimensional acceleration of one’s bodily movement. If listening contexts influenced the audience’s bodily movement, this aspect could interfere with the audience’s physiological reactions. To explore such a possibility, we calculated the mean acceleration of each audience member’s for each piece. We performed a paired *t*-test for each piece by using Bonferroni’s correction, showing no significant differences between the live and the recorded conditions (Table 1). This indicates that the acceleration of the audience’s bodily movement did not differ between the listening contexts.

**Table 1:** The mean values, the standard deviations, and 95% confidence intervals of audience’s acceleration (mG) for each piece in each condition (*N* = 37). Results of paired *t*-tests between the listening conditions were shown in the right columns.

	Live Condition		Recorded Condition		Paired *t*-test		
Piece	*M*(*SD*)	95% CI	*M*(*SD*)	95% CI	*t*(36)	*p*	$\eta_{p}^{2}$
Resting Phase	11.36 (1.73)	[10.77, 11.94]	11.39 (1.86)	[10.76, 12.02]	0.07	0.94	0.02
Listening Phase (Overall Mean)	11.62 (1.27)	[11.20, 12.05]	11.71 (1.36)	[11.26, 12.17]			
B24	10.86 (1.41)	[10.39, 11.34]	11.09 (1.79)	[10.49, 11.70]	0.71	0.49	0.01
B15	11.65 (1.90)	[11.01, 12.29]	11.74 (1.84)	[11.12, 12.36]	0.20	0.84	< 0.01
Dreaming	12.02 (2.11)	[11.30, 12.73]	12.18 (2.15)	[11.46, 12.91]	0.34	0.73	< 0.01
Soaring	12.27 (2.91)	[11.29, 13.26]	11.91 (1.94)	[11.25, 12.56]	0.57	0.56	0.01
Girl	11.34 (1.90)	[10.70, 11.99]	12.02 (2.01)	[11.35, 12.69]	1.55	0.13	0.06
Arabesque	11.59 (1.72)	[11.01, 12.17]	11.33 (1.80)	[10.72, 11.93]	0.73	0.47	0.02

Next we examined effects of the audience member’s age and extracurricular musical training by computing Pearson’s correlation coefficients with each of the electrocardiogram parameters (i.e., HR, HF/TF, ln(LF/HF)) (see S1 Table). Because the analyses were performed 14 times for each parameter (i.e., (6 pieces + the resting phase) × 2 listening contexts), the significance level was adjusted by Bonferroni’s correction (Overall *α* = 0.10, subset *α* = 0.10/14 ≈ 0.007). Results showed no significant correlations. We also examined effects of the audience’s and the performer’s sex, showing neither significant main effects nor interaction (*α* = 0.007, see S2 Table). Finally we examined effects of the individual performer on the audience’s heart-rate activities, but no significant effects were found (*α* = 0.007, see S3 Table). These analyses indicate that the age, the degree of musical training, the sex of either the audience or the performer, and the performer’s individuality did not affect the heart-rate activities in the present study.

There were not significantly different heart-rate activities during the resting phase between the live and the recorded conditions (see the first row for each parameter in Table 2): *t*(36) = 0.99, *p* = 0.83, $\eta_{p}^{2}<0.01$ (HR), *t*(36) = 0.40, *p* = 0.34, $\eta_{p}^{2}=0.03$ (HF/TF), *t*(36) = 0.44, *p* = 0.34, $\eta_{p}^{2}=0.03$ (ln(LF/HF)). In the following analyses, therefore, we used pooled raw data, rather than difference values from the measurements during the resting phase, though both cases generated similar tendencies according to our inspection.

**Table 2:** The mean values, the standard deviations, and 95% confidence intervals of HR (a), the HF/TF ratio (b), and the natural logarithm of the LF/HF ratio (c) for each piece in each condition (*N* = 37).

	Live Condition		Recorded Condition	
Piece	*M*(*SD*)	95% CI	*M*(*SD*)	95% CI
(a) HR (beats/m)				
Resting Phase	79.63 (12.85)	[75.35, 83.92]	80.63 (17.38)	[74.83, 86.42]
Listening Phase (Overall Mean)	76.82 (11.99)	[72.82, 80.82]	79.80 (19.22)	[73.39, 86.21]
B24	75.73 (11.34)	[71.95, 79.51]	80.28 (19.50)	[73.78, 86.79]
B15	76.78 (13.23)	[72.37, 81.19]	81.28 (19.22)	[74.87, 87.69]
Dreaming	76.64 (13.39)	[72.18, 81.10]	81.01 (22.24)	[73.59, 88.42]
Soaring	78.00 (12.09)	[73.97, 82.03]	79.15 (20.79)	[72.21, 86.08]
Girl	76.14 (12.86)	[71.85, 80.43]	79.30 (18.74)	[73.05, 85.54]
Arabesque	77.61 (13.05)	[73.26, 81.96]	77.79 (19.88)	[71.16, 84.42]
(b) HF/TF				
Resting Phase	46.36 (20.82)	[39.42, 53.30]	43.12 (16.18)	[37.73, 48.52]
Listening Phase (Overall Mean)	49.96 (9.66)	[46.74, 53.18]	43.83 (14.45)	[39.01, 48.64]
B24	47.85 (20.37)	[41.05, 54.64]	43.89 (20.20)	[37.16, 50.63]
B15	50.03 (22.77)	[42.44, 57.63]	45.10 (16.99)	[39.43, 50.76]
Dreaming	52.78 (24.49)	[44.61, 60.94]	46.05 (20.83)	[39.10, 52.99]
Soaring	49.03 (23.01)	[41.36, 56.70]	43.90 (16.93)	[38.26, 49.55]
Girl	50.46 (22.92)	[42.82, 58.11]	38.49 (17.81)	[32.55, 44.42]
Arabesque	49.61 (22.57)	[42.09, 57.14]	45.54 (19.56)	[39.02, 52.07]
(c) ln(LF/HF)				
Resting Phase	0.15 (0.99)	[-0.18, 0.48]	0.31 (0.75)	[0.06, 0.56]
Listening Phase (Overall Mean)	0.03 (0.51)	[-0.14, 0.20]	0.27 (0.69)	[0.05, 0.50]
B24	0.10 (0.96)	[-0.22, 0.42]	0.31 (0.96)	[-0.01, 0.63]
B15	0.03 (1.17)	[-0.36, 0.42]	0.20 (0.79)	[-0.06, 0.46]
Dreaming	-0.12 (1.24)	[-0.53, 0.30]	0.12 (1.06)	[-0.23, 0.48]
Soaring	0.07 (1.15)	[-0.31, 0.46]	0.26 (0.75)	[0.01, 0.51]
Girl	0.02 (1.23)	[-0.39, 0.43]	0.55 (0.85)	[0.26, 0.83]
Arabesque	0.06 (1.16)	[-0.32, 0.45]	0.21 (0.93)	[-0.10, 0.52]

### Effects of listening context, composer, and tempo on the audience’s heart rate and heart-rate variability

Table 2 shows the mean, the standard deviation, and the 95% confidence interval for each parameter per piece between the two listening conditions. Because heart-rate data for each participant generally correlate between experimental conditions, the sphericity assumption in within-subject analysis of variance are not satisfied [14]. In the analyses reported below, we corrected the degrees of freedom by Greenhouse and Geisser’s method, as recommended by Keselman and Rogan [19].

**Fig 1:**
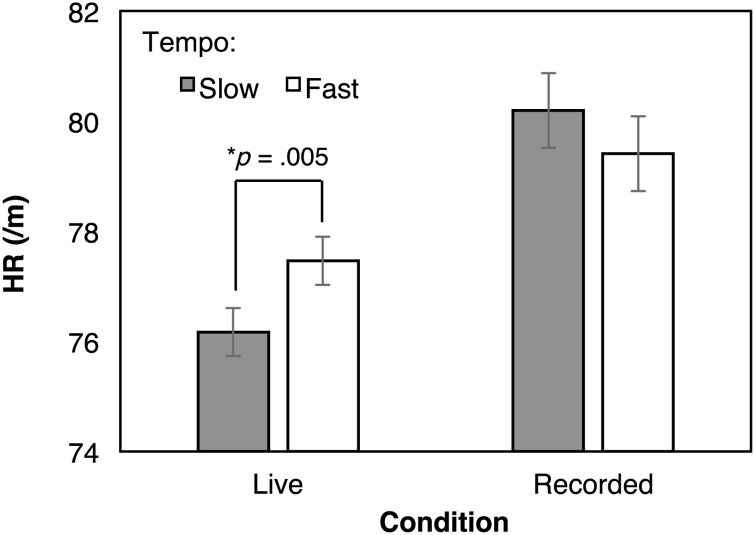
The mean values of the audience’s heart rate (beats per minute) for the two-way interaction between the listening context and the tempo. Error bars indicate standard errors. The *p*-value indicates a significant difference confirmed by the post-hoc *t*-test.

We conducted a 2 (context) × 3 (composer) × 2 (tempo) within-subject analysis of variance for each parameter (Table 3). For HR, only a two-way interaction between listening context and tempo was significant. Fig 1 shows the mean values for the two-way interaction. The post-hoc *t*-tests (using Shaffer’s modified sequentially rejective Bonferroni procedure) showed that HR was significantly greater for the faster (*M* = 77.46, *SD* = 12.69) than the slower pieces (*M* = 76.17, *SD* = 12.45) in the live condition (*p* = 0.005, $\eta_{p}^{2}=0.20$), but not in the recorded condition (*p* = 0.25, $\eta_{p}^{2}=0.04$). This suggests that the audience’s heartbeat changes along with the tempo of music only during live performance.

**Table 3:** The results of 2 (context) × 3 (composer)× 2 (tempo) within-subject analyses of variance for HR (a), the HF/TF ratio (b), and the natural logarithm of the LF/HF ratio (c). The degrees of freedom were adjusted by Greenhouse-Geisser method.

Source	*df*1	*df*2	*F*	*p*	$\eta_{p}^{2}$
(a) HR					
A: Context	1	36	1.38	0.25	0.04
B: Composer	1	71	1.10	0.34	0.03
C: Tempo	1	36	0.40	0.53	0.01
A × B	1	68	2.16	0.13	0.06
B × C	1	69	0.44	0.64	0.01
C × A	1	36	6.38	0.02*	0.15
A × B × C	1	67	1.22	0.30	0.03
(b) HF/TF					
A: Context	1	36	4.73	0.04*	0.12
B: Composer	1	71	0.31	0.73	0.01
C: Tempo	1	36	0.13	0.72	< 0.01
A × B	1	71	0.33	0.72	0.01
B × C	1	65	1.19	0.31	0.03
C × A	1	36	1.32	0.26	0.04
A × B × C	1	71	0.59	0.56	0.02
(c) ln(LF/HF)					
A: Context	1	36	3.41	0.07^†^	0.09
B: Composer	1	71	0.49	0.61	0.01
C: Tempo	1	36	0.08	0.78	< 0.01
A × B	1	71	0.23	0.79	0.01
B × C	1	66	1.38	0.26	0.04
C × A	1	36	1.87	0.18	0.05
A × B × C	1	71	0.47	0.63	0.01

* *p* < .05,

^†^ *p* < .10

The HF/TF ratio showed a significant main effect of context (Table 3). HF/TF in the live condition was significantly greater than that in the recorded condition (see Table 2). This suggests that the audience’s vagal nerve is activated more in the live than the recorded condition.

The LF/HF ratio was less in the live than the recorded condition (see Table 2); main effect of context was approaching significant (Table 3). This appears to imply that the contribution of the symapthetic against the vagal nerve activity tended to decrease in the live condition.

## Discussion

The present results showed that during live performance, the vagal nerve activity (i.e., HF/TF) increased and the sympathovagal balance (i.e., LF/HF) tended to decrease regardless of the piece. Thus, the pianist’s live performance appears to have led the audience’s nerve activities toward induction of relaxation or reduction of anxiety. This finding implies that sharing time and place with a performer is not awkward but normal (or spontaneous) for the audience, supporting that such a social context facilitates stress reduction during a cognitive task [9] and music listening [20]. From another point of view, a high vagal activity is associated with the stable visual attention to objects (e.g., [21]). The higher vagal activity in the live than in the recorded condition may also imply that the audience was more attentive to live performance.

In contrast with the present study, Chapados and Levitin [4] reported an increase of the autonomic nerve activity in experiencing the video of Stravinsky’s clarinet piece. This discrepancy between the two studies may derive from the availability of a performer’s body movements. More specifically, the body movements of pianists (centered at the piano chair) are more limited than the whole body movements of clarinetists, so that the audience’s visual attention to the performance can be more stable for the piano than for the clarinet performance. If the vagal nerve is activated by “stable” visual attention (e.g., [21]), it is understandable that the piano and the clarinet live performances generate opposite heart-rate responses.

We also showed that the audience’s heart rate changed in accordance with the tempo of music only in the live condition. One of the features of biological oscillators such as heart beat and respiration is to synchronize with, or to be entrained by, external inputs [22, 23]. Some researchers have shown that the listener’s heart beat tends to be entrained by the tempo of music (e.g., [24]) while others have reported no specific relations between them (e.g., [25]). The recent review [26] shows that there is no evidence for entrainment of the heart rate to musical beats in listening to music, at least, via sound without visual information. The present result indicates that the listening context contributes another factor to be considered in this controversy: Live listening facilitates entrainment between tempo and heart rate while listening to recorded music does not.

There are at least two possible reasons for the present contextual effect. First, the audience’s heart rate may be influenced by the performer’s body movement that tends to reflect the tempo of music [27]. Second, the live context may have generated a social interaction allowing the performer and the audience to share the same musical moments, something similar to a conversation in which the heart rates of speakers tend to be synchronized [28]. This “sharing the musical moments” may have resulted in the audience’s heart rates varying in accordance with the tempo of music, which, in turn, could explain why live performances sound more artistic and expressive, as well as why their affective nuances perceived by the audience are closer to those interpreted by the performer than recorded ones [29].

In sum, we have revealed effects of live performance on the audience’s physiological reaction. The audience’s vagal nerve is activated in the live context, suggesting that live performance reduces stress and induces attention in the audience as compared with the recorded performance. The physiological entrainment by musical tempo can be facilitated only during live performance. These contextual effects, however, need to be interpreted with caution, for we sacrificed the controlled design by prioritizing ecological validity. We cannot deny that all the contextual effects on the audience’s heart-rate activities in the present study are confounded with a few variables such as the order of listening context, the repeated presentation of the same performance, and the sound quality (live versus loud speakers). In order to verify the causal function of “live” performance, we need to sort them out through a series of controlled experiments. For example, replication of the present study by utilizing between-subjects design is one way to tap into the issue of the order effect. Installing two controlled groups—one listening to live performances twice and the other to recorded performances twice—may be another way to resolve the aforementioned confounding variables. Using both an acoustic and an electronic instrument in live performance may be effective in examining an effect of the sound quality.

Other aspects of music listening that were missing from the present study were the audience’s subjective impressions about their own stress levels as well as valence and arousal of performances, which might be related to heart-rate activities [26]. Moreover, studying the audience’s actual movement such as their head movement (rather than its accelerations) may help us understand the nature of visual attention during live performance in more depth [30]. Relations among performance parameters (e.g., tempo, dynamics, the pianist’s body movement), the audience’s subjective and behavioral reactions, and the audience’s physiological responses need to be mapped together in order to capture the entire picture of the performer-and-audience communication in live performance.

## Supporting Information

S1 Table Pearson’s correlation coefficients (*r*) between each parameter and each of the participant’s age and the years of musical training. All the correlations were insignificant (*df* = 35, *α* = 0.007 with Bonferroni’s correction).
(PDF)

S2 Table The results of 2 (audience sex) × 2 (performer sex) between-subjects analyses of variance for each parameter (*α* = .007). (PDF)

S3 Table The results of one-way between-subjects analyses of variance that examined effects of the individual performer on the electrocardiogram parameters for each piece (*α* = .007). (PDF)
