# Supplementary material for: How Live Performance Moves the Human Heart
Source: PLoS One. 2016 Apr 22;11(4):e0154322. doi: 10.1371/journal.pone.0154322 (PMC4841601; doi:10.1371/journal.pone.0154322)
Supplement: S1 Table — All the correlations were insignificant (df = 35, α = 0.007 with Bonferroni’s correction). (PDF) [file pone.0154322.s001.pdf]

	Age		Musical Training	
Piece	Live	Recorded	Live	Recorded
		HR (beats/m)		
Resting Phase	-0.05	-0.24	-0.25	-0.09
Listening Phase				
B24	-0.08	0.11	0.04	0.13
B15	0.03	0.22	0.07	0.01
Dreaming	-0.12	0.19	0.08	0.12
Soaring	-0.15	0.10	0.11	0.18
Girl	-0.17	0.17	0.01	0.00
Arabesque	-0.12	0.15	-0.02	0.13
		HF/TF		
Resting Phase	-0.15	-0.05	0.13	0.08
Listening Phase				
B24	-0.11	-0.23	-0.02	0.06
B15	0.19	-0.09	-0.04	0.11
Dreaming	-0.14	-0.01	-0.15	0.19
Soaring	-0.14	0.25	-0.35	0.06
Girl	-0.06	-0.19	-0.23	0.11
Arabesque	-0.02	-0.14	0.03	0.16
		ln(LF/HF)		
Resting Phase	0.18	0.03	-0.13	-0.06
Listening Phase				
B24	0.10	0.21	0.04	-0.01
B15	-0.18	0.10	-0.02	-0.11
Dreaming	0.17	0.02	0.17	-0.17
Soaring	0.13	-0.26	0.35	-0.04
Girl	0.10	0.18	0.29	-0.13
Arabesque	0.02	0.13	0.01	-0.14
