# Supplementary material for: How Live Performance Moves the Human Heart
Source: PLoS One. 2016 Apr 22;11(4):e0154322. doi: 10.1371/journal.pone.0154322 (PMC4841601; doi:10.1371/journal.pone.0154322)
Supplement: S2 Table — (PDF) [file pone.0154322.s002.pdf]

Piece	The Audience's Sex			The Performer's Sex			Two-way Interaction		
	F(1,33)	p	η_p^2	F(1,33)	p	η_p^2	F(1,33)	p	η_p^2
					HR				
**Live Condition**									
Resting Phase	0.43	0.52	0.01	0.80	0.38	0.02	0.01	0.99	< 0.01
Listening Phase									
B24	3.27	0.08	0.09	3.06	0.09	0.08	1.87	0.18	0.05
B15	0.58	0.45	0.02	1.54	0.22	0.04	0.09	0.77	< 0.01
Dreaming	1.70	0.20	0.05	0.84	0.37	0.02	1.13	0.30	0.03
Soaring	2.59	0.12	0.07	2.52	0.12	0.07	0.14	0.71	< 0.01
Girl	1.26	0.27	0.04	0.05	0.82	< 0.01	0.00	1.00	< 0.01
Arabesque	3.03	0.09	0.08	0.63	0.43	0.02	0.23	0.64	0.01
**RecordedCondition**									
Resting Phase	0.59	0.45	0.02	0.05	0.82	< 0.01	1.66	0.21	0.05
Listening Phase									
B24	3.08	0.09	0.09	7.37	0.01	0.18	2.14	0.15	0.06
B15	1.89	0.18	0.06	6.12	0.02	0.16	0.20	0.66	0.01
Dreaming	3.43	0.07	0.10	2.90	0.10	0.08	1.59	0.22	0.05
Soaring	4.68	0.04	0.13	6.87	0.01	0.17	2.73	0.11	0.08
Girl	5.24	0.03	0.14	4.83	0.04	0.13	0.19	0.66	0.01
Arabesque	4.67	0.04	0.13	6.49	0.02	0.16	2.18	0.15	0.06
					HF/TF				
**Live Condition**									
Resting Phase	0.20	0.66	0.01	1.82	0.19	0.05	0.79	0.38	0.02
Listening Phase									
B24	0.53	0.47	0.02	1.01	0.32	0.03	0.85	0.36	0.03
B15	0.01	0.94	< 0.01	1.73	0.20	0.05	0.09	0.77	< 0.01
Dreaming	0.24	0.62	0.01	3.16	0.08	0.09	0.84	0.37	0.02
Soaring	0.07	0.80	< 0.01	1.37	0.25	0.04	0.35	0.56	0.01
Girl	0.07	0.80	< 0.01	0.31	0.58	0.01	0.66	0.42	0.02
Arabesque	0.27	0.60	0.01	3.40	0.07	0.09	0.02	0.89	< 0.01
**RecordedCondition**									
Resting Phase	1.64	0.21	0.05	2.90	0.10	0.08	0.64	0.43	0.02
Listening Phase									
B24	0.03	0.86	< 0.01	2.18	0.15	0.06	1.08	0.31	0.03
B15	0.01	0.93	< 0.01	1.91	0.18	0.05	0.81	0.37	0.02
Dreaming	0.32	0.58	0.01	0.01	0.91	< 0.01	1.16	0.29	0.03
Soaring	0.01	0.99	< 0.01	0.01	0.93	< 0.01	1.12	0.30	0.03
Girl	0.13	0.72	< 0.01	1.38	0.25	0.04	8.24	0.01	0.20
Arabesque	2.91	0.10	0.08	2.98	0.09	0.08	6.85	0.01	0.17
					ln(LF/HF)				
**Live Condition**									
Resting Phase	0.21	0.65	0.01	2.06	0.16	0.06	0.97	0.33	0.03
Listening Phase									
B24	0.63	0.43	0.02	0.99	0.33	0.03	1.08	0.31	0.03
B15	0.06	0.81	< 0.01	1.80	0.19	0.05	0.01	0.91	< 0.01
Dreaming	0.14	0.71	< 0.01	3.13	0.09	0.09	0.52	0.48	0.02
Soaring	0.05	0.83	< 0.01	1.07	0.31	0.03	0.11	0.74	< 0.01
Girl	0.26	0.61	0.01	0.68	0.42	0.02	0.33	0.57	0.01
Arabesque	0.10	0.75	< 0.01	3.28	0.08	0.09	0.01	0.98	< 0.01
**RecordedCondition**									
Resting Phase	1.68	0.20	0.05	2.94	0.10	0.08	0.71	0.40	0.02
Listening Phase									
B24	0.69	0.41	0.02	0.28	0.60	0.01	1.05	0.31	0.03
B15	0.01	0.97	< 0.01	1.32	0.26	0.04	1.08	0.31	0.03
Dreaming	0.01	0.97	< 0.01	0.03	0.87	< 0.01	0.96	0.33	0.03
Soaring	0.14	0.71	< 0.01	0.01	0.97	< 0.01	1.55	0.22	0.04
Girl	2.45	0.13	0.07	3.26	0.08	0.09	6.33	0.02	0.16
Arabesque	0.14	0.71	< 0.01	1.35	0.25	0.04	8.47	0.01	0.20
