# Supplementary material for: How Live Performance Moves the Human Heart
Source: PLoS One. 2016 Apr 22;11(4):e0154322. doi: 10.1371/journal.pone.0154322 (PMC4841601; doi:10.1371/journal.pone.0154322)
Supplement: S3 Table — (PDF) [file pone.0154322.s003.pdf]

| | Live Condition | | | Recorded Condition | | |
|---|---|---|---|---|---|---|
| Piece | $F(1,33)$ | $p$ | $\eta^2$ | $F(1,33)$ | $p$ | $\eta^2$ |
| | | | HR | | | |
| Resting Phase | 1.66 | 0.17 | 0.25 | 2.36 | 0.05 | 0.32 |
| Listening Phase | | | | | | |
| B24 | 1.15 | 0.36 | 0.19 | 1.94 | 0.11 | 0.28 |
| B15 | 1.30 | 0.29 | 0.21 | 1.44 | 0.23 | 0.22 |
| Dreaming | 0.88 | 0.52 | 0.15 | 1.56 | 0.19 | 0.24 |
| Soaring | 1.79 | 0.14 | 0.26 | 2.05 | 0.09 | 0.29 |
| Girl | 0.76 | 0.61 | 0.13 | 1.38 | 0.26 | 0.22 |
| Arabesque | 0.91 | 0.50 | 0.15 | 1.62 | 0.18 | 0.24 |
| | | | HF/TF | | | |
| Resting Phase | 1.86 | 0.12 | 0.27 | 0.50 | 0.80 | 0.09 |
| Listening Phase | | | | | | |
| B24 | 1.43 | 0.24 | 0.22 | 0.76 | 0.60 | 0.13 |
| B15 | 1.52 | 0.20 | 0.23 | 0.72 | 0.63 | 0.13 |
| Dreaming | 1.21 | 0.33 | 0.19 | 2.17 | 0.07 | 0.30 |
| Soaring | 0.61 | 0.72 | 0.11 | 0.31 | 0.93 | 0.06 |
| Girl | 1.71 | 0.15 | 0.25 | 0.39 | 0.88 | 0.07 |
| Arabesque | 2.71 | 0.03 | 0.35 | 0.80 | 0.58 | 0.14 |
| | | | ln(LF/HF) | | | |
| Resting Phase | 1.96 | 0.10 | 0.28 | 0.52 | 0.79 | 0.09 |
| Listening Phase | | | | | | |
| B24 | 1.43 | 0.24 | 0.22 | 0.81 | 0.57 | 0.14 |
| B15 | 1.71 | 0.15 | 0.25 | 0.58 | 0.74 | 0.10 |
| Dreaming | 1.42 | 0.24 | 0.22 | 1.67 | 0.16 | 0.25 |
| Soaring | 0.59 | 0.74 | 0.11 | 0.37 | 0.89 | 0.07 |
| Girl | 1.88 | 0.12 | 0.27 | 0.45 | 0.84 | 0.08 |
| Arabesque | 2.79 | 0.03 | 0.36 | 0.92 | 0.50 | 0.16 |
